# Thermoneutral housing temperature regulates T-regulatory cell function and inhibits ovabumin-induced asthma development in mice

**DOI:** 10.1038/s41598-017-07471-7

**Published:** 2017-08-02

**Authors:** Wenjing Liao, Libo Zhou, Xiaolong Zhao, Lijuan Song, Yingshen Lu, Nanshan Zhong, Pingchang Yang, Baoqing Sun, Xiaowen Zhang

**Affiliations:** 1 State Key Laboratory of Respiratory Disease, Department of Otolaryngology-Head and Neck Surgery, Guangzhou, 510120 China; 2 Laboratory of ENT-HNS Disease, First Affiliated Hospital, Guangzhou Medical University, Guangzhou, 510120 China; 3 Department of Allergy and Clinical Immunology, State Key Laboratory of Respiratory Disease, First Affiliated Hospital, Guangzhou Medical University, Guangzhou, 510120 China; 4 National Clinical Research Center, Guangzhou, 510120 China

## Abstract

The change in ambient temperature is one of the risk factors for the aggravation of bronchial asthma (BA). Yet, whether the ambient temperature influences the immune functions associated with allergic asthma remains unknown. In this study, we treated asthmatic mice with standard temperature (ST, 20 °C) or thermoneutral temperature (TT, 30 °C). The results showed that the airway inflammatory cell counts in bronchoalveolar lavage fluid (BALF) and airway hyperresponsiveness (AHR) were significantly reduced in the mice treated with TT as compared with the mice treated with ST. The imbalance of Th1/Th2 response in the lung was improved following housing the mice at TT. In addition, the pulmonary Treg cells were increased in asthmatic mice after TT treatment. The temperature stress (29 °C and 41 °C) drove naïve CD4T cells towards Th2 cells. Our data demonstrate that the change of ambient temperature was a risk factor to aggravate experimental asthma.

## Introduction

The effects of weather change on aggravating bronchial asthma (BA) have been noticed^1^. It is suggested the cold air imposes considerable effects on compromising the lung functions in individual asthmatics as well as susceptible populations^2^. About 70% of patients with asthma reported that the cold air was one of the important factors in triggering difficulty breathing, which affects about 37% of asthma patient outdoor activities during winter^3^. It is suggested that cold air inhalation may trigger airway inflammatory cell infiltration and epithelial barrier damage among normal and BA subjects^2, 4, 5^. Yet, the underlying mechanism of these clinical phenomena needs to be further investigated.

Mouse models are widely used in research to investigate disease mechanism. Previous reports indicated that the standard laboratory temperature (ST, 20 °C) imposed considerably effects on mouse living as compared to the thermoneutral temperature (TT, 30 °C)^6–9^. Published data indicate that cold stress can alter mouse’s physiology, including behavioral thermoregulation^10^, metabolic rate^11^, sympathetic activity^6^, fatty acid oxidation, energy homeostasis^12, 13^, and immune responses^7–9^. Whether the ambient temperature change influences the immunity associated with BA in mice remains to be determined.

In this study, an OVA-induced asthmatic mouse model was developed. The mice were treated with TT or ST. The immune profiles of these mice were assessed. The results showed that TT markedly attenuated the asthma symptoms, improved the Th1/Th2 balance and increased the development of Tregs.

## Results

### TT attenuates AHR and inflammatory cell influx and reduces pulmonary histopathology changes in asthmatic mice

**Figure 1:**
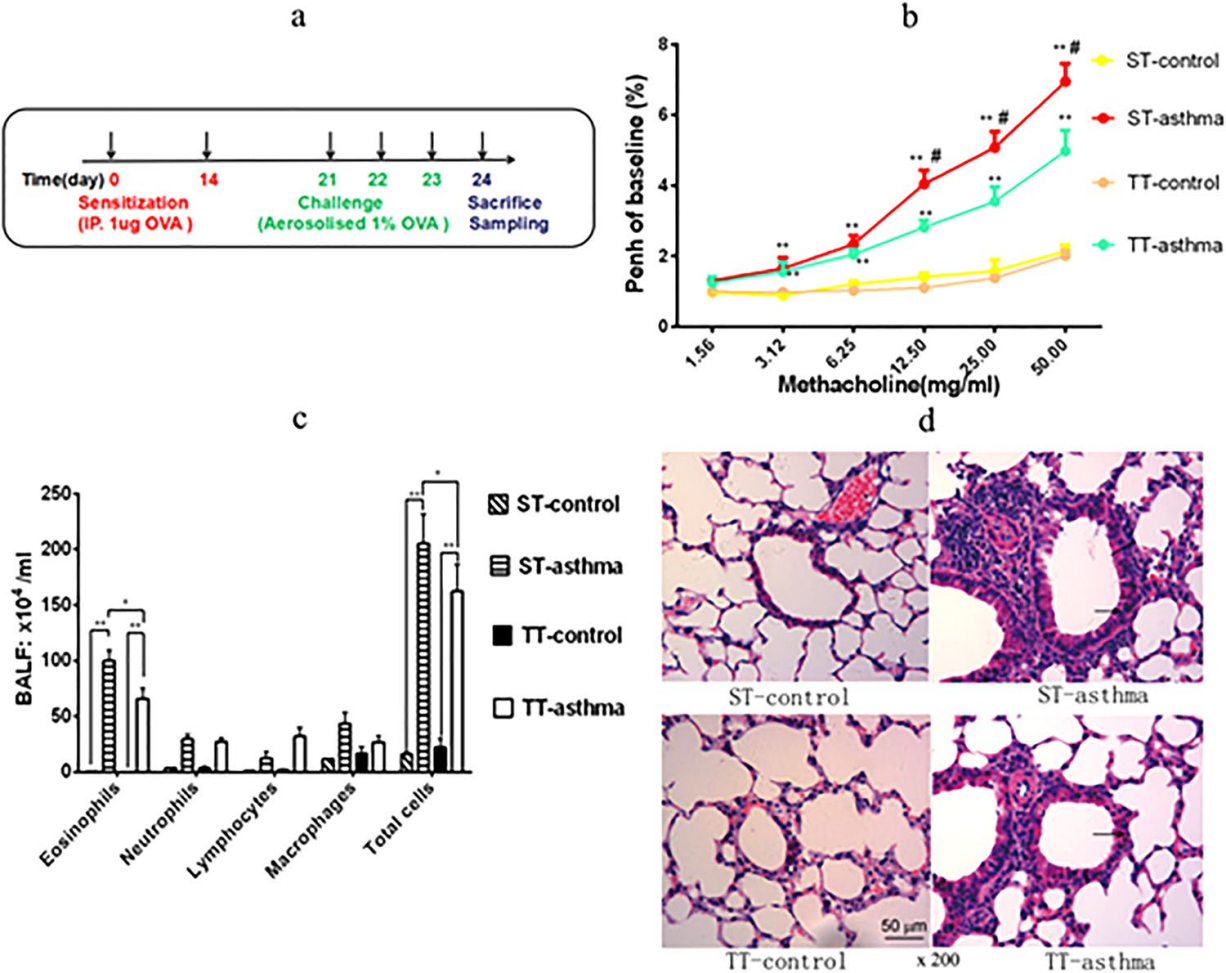
Effects of the changing ambient temperature on asthma mice. (**a**) The experimental procedures using to develop an asthma mouse model was generated by using OVA sensitization and challenge. (**b**) The AHR in mice after being inhaled Mch was measured recorded using flow plethysmography. (**c**) Total and differential cells numbers in BALF that were determined by microscopyicobservation. The data were averaged from for five randomly selected fields of each mouse. (**d**) The representative photomicrographs (200×)of lung sections are shown. Black arrows indicate areas of peribronchiolar inflammatory cell influx and edema. Black bars indicate the amount of bronchiolar remodeling. ST: standard temperature (20 °C); TT: thermoneutral temperature (30 °C). The data presented are mean ± SEM. ** denotes P < 0.01 (ST-control vs. ST-asthma or TT-control vs.TT-asthma); # denotes P < 0.05 (TT-asthma vs. ST-asthma) in Fig. 1b. * or ** denotes P < 0.05, P < 0.01 respectively between two groups in Fig. 1c,d (n = 6 mice per group).

In a mice model of allergic asthma (Fig. 1a), twenty-four hours after the last challenge with specific antigens, the mice were placed in the chambers for AHR evaluation at the same living temperatures, and then sacrificed for subsequent BALF analysis. The AHR was assessed by measuring an enhanced pause (Penh) for baselines. The Penh% values in the asthma groups were significantly higher than those in the control groups (p < 0.01, Fig. 1b). The Penh% in the TT-asthma group was lower compared with that of the ST- asthma group (Fig. 1b). In BALF, the number of eosinophils and total inflammatory cells were significantly more in the asthma group compared with those in the naïve control group (p < 0.01, Fig. 1c). Exposure of asthma mice to TT markedly reduced the airway total inflammatory cells and eosinophils as compared to those asthma mice at ST (p < 0.05, Fig. 1c). The histopathology of lung showed perivascular and peribronchiolar eosinophilia, oedema and epithelial damage in the asthmatic mice, which was markedly attenuated in the TT group (Table 1, and Fig. 1d).

**Table 1:** Histopathological scoring of inflammatory change in the lungs of mice.

	ST-control	ST-asthma	TT-control	TT-asthma
Peribronchiolar eosinophilia	0.33 ± 0.52*	3.16 ± 0.75	0.16 ± 0.40*	2.11 ± 0.63^#^
Perivascular eosinophilia	0.16 ± 0.40*	1.83 ± 0.75	0.33 ± 0.82*	1.33 ± 0.52
Oedema	0.33 ± 0.52*	3.52 ± 0.55	0.16 ± 0.40*	1.83 ± 0.75^#^
Epithelial damage	0.16 ± 0.40*	3.51 ± 1.05	0.33 ± 0.82*	3.01 ± 0.89

Inflammatory changes were graded by histopathological assessment using a semiquantitative scale of 0–5 (Table 2). ST: standard temperature (20 °C); TT: thermoneutral temperature (30 °C). The data presented are mean ± SEM. * denotes P < 0.05 (ST-control vs. ST-asthma or TT-control vs.TT-asthma); ^#^denotes P < 0.05 (TT-asthma vs. ST-asthma) (n = 6 mice per group).

### The effects of changing ambient temperature on expression of IL-4, IL-13, IL10 and IFN-γ and OVA specific IgE in asthma mice

**Figure 2:**
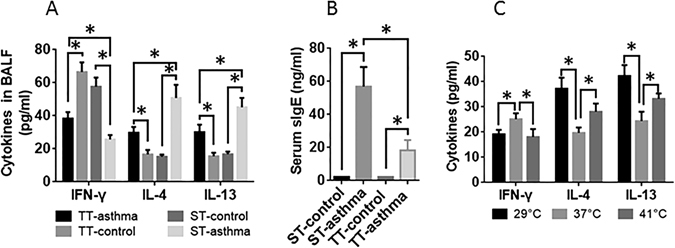
The effects of ambient temperature on IFN-γ, IL-4, IL-13 and serum OVA specific IgE. The BALF and sera were collected from the mice and analyzed by ELISA. The bars indicate the cytokine levels in the BALF (**A**) and the antigen-specific IgE in the serum (**B**). ST: standard temperature (20 °C); TT: thermoneutral temperature (30 °C). Control: Naïve mice. Asthma: Asthma mice. (**C**) Spleen cells were cultured at the indicated temperature for 48 h in the presence of ionomycin. The data are presented as mean ± SEM. *P < 0.01 (t test). Each group consists of 6 mice.

We next assessed the effects of changing the ambient temperature on the cytokine profile in the lung of asthma mice. The results showed that treating asthma mice with TT significantly suppressed the levels of IL-4 and IL-13, and increased the IFN-γγ levels in the BALF, as compared with the data of asthma mice treated with ST (Fig. 2A). In addition, treating asthma mice with TT also suppressed the serum antigen-specific IgE as compared to that treated with ST (Fig. 2B). The results demonstrate that treating asthma mice with TT can suppress the levels of Th2 cytokines in the lung and the serum specific IgE in asthma mice.

### The effect of cell culture temperature on cytokine production bysplenocytes *in vitro*

To further assess the effects of changing temperature on the regulation of cytokine production by CD4^+^ T cells, naïve CD4^+^ T cells were isolated from the spleen of naïve mice. The cells were cultured at 29 °C, or 37 °C, or 41 °C, respectively, in the presence of inomycin for 48 h. The supernatant was analyzed by ELISA. The results showed that, as compared to cells cultured at 37 °C, CD4^+^ T cells cultured at either 29 °C or 41 °C produced less IFN-γγ and more IL-4 and IL-13 (Fig. 2C). The results demonstrate that changing the ambient temperature results skewed Th2 polarization

### The subsets of Th1/Th2 and regulatory T (Treg) cells can be altered by ambient temperature change

**Figure 3:**
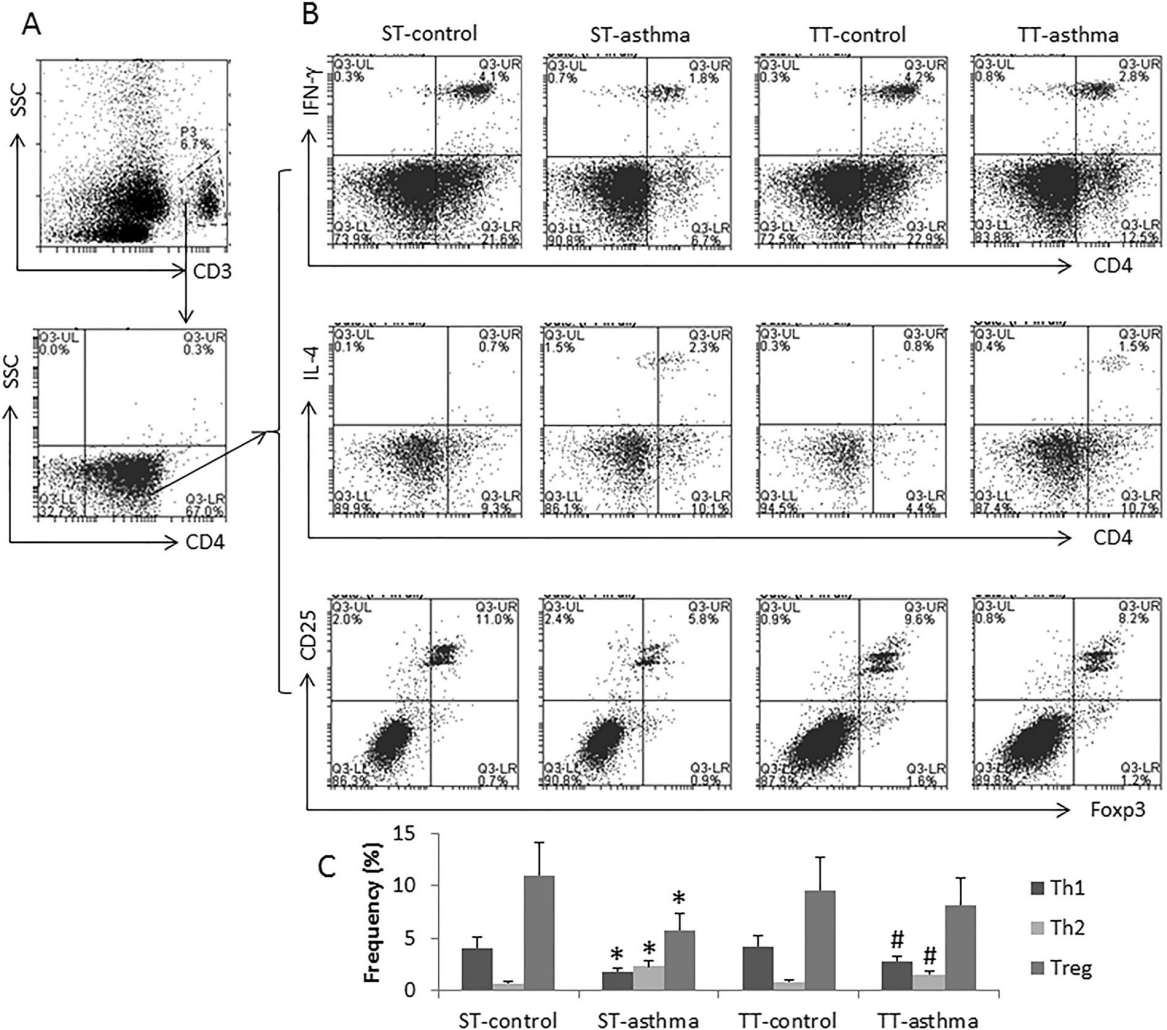
Effects of changing ambient temperature on Th1/Th2 cell and Treg cell populations in the spleen. Spleen cells were prepared from each mouse after treating with ST or TT. The cells were analyzed by flow cytometry. (**A**) The dot plots show the CD3^+^ and CD4^+^ T cells were gated. (**B**) The dot plots show the frequency of IFN-γ^+^, IL-4^+^ and IL-13^+^ cells, respectively, in the gated CD4^+^ T cells of panel A. C, the bars show the summarized data of panel B. The data of bars are presented as mean ± SEM. *p < 0.05 (t test), compared to the ST-control group. #p < 0.05, compared to the TT-control group. Each group consists of 6 mice.

Flow cytometric analysis was performed to evaluate the impact of ambient temperature change on the subsets of CD4^+^ T cells. The representative dot plots of mouse spleen CD4^+^ T cells are presented in Fig. 3. The results showed that the frequency of Th1 cell and Treg was lower, the frequency of Th2 cells was higher in asthma mice treated with ST than that in control mice, which was markedly reversed by treating mice with TT. The results demonstrate that TT can redress the Th1/Th2 imbalance and enhances Treg development in mice with asthma.

### The effects of ambient temperature on Foxp3, GATA-3 and Tbet mRNA expression

**Figure 4:**
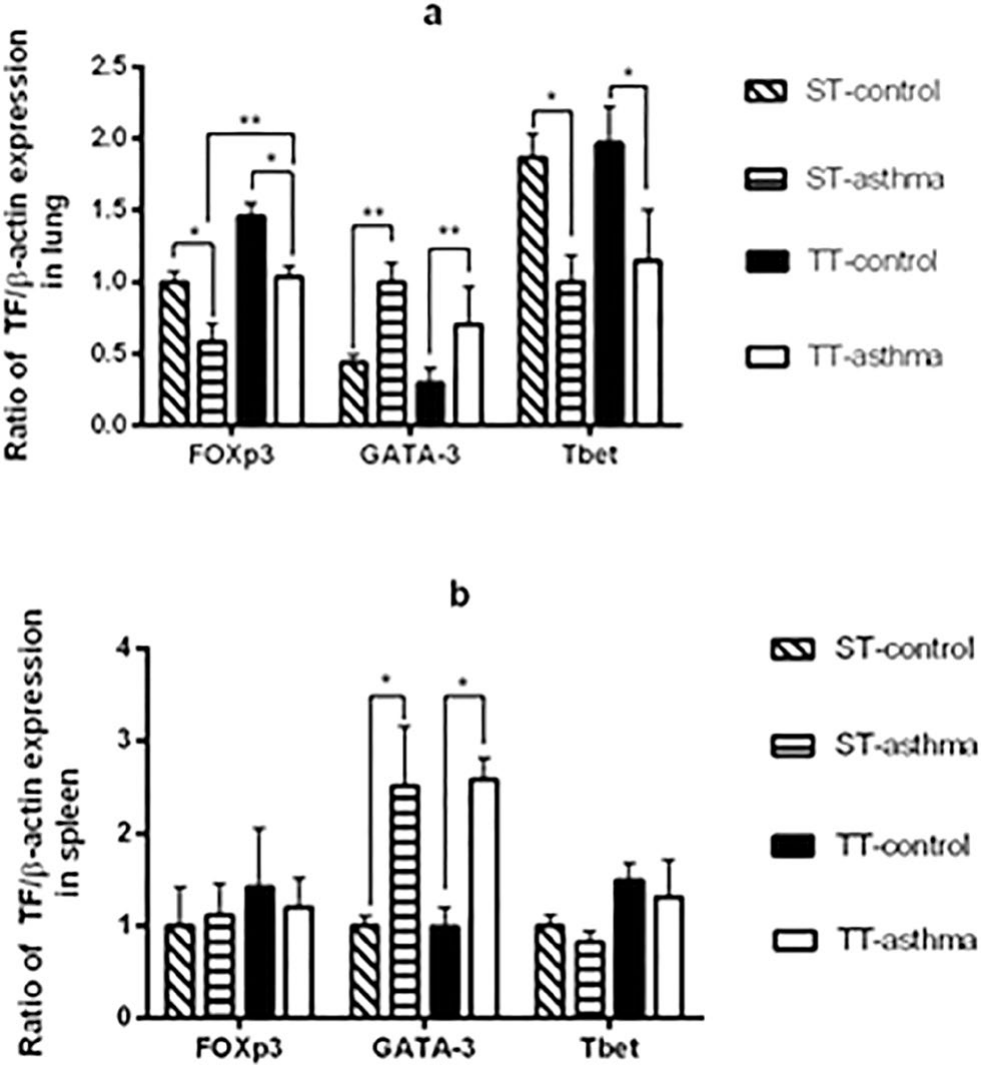
Effects of ambient temperature on Foxp3, T-bet, and GATA3 expression in the lung and spleen. The mRNA expression of Foxp3, T-bet, and GATA3 in the lung tissue (**a**) and spleen tissue (**b**) were detected by RT-PCR. ST: standard temperature (20 °C); TT: thermoneutral temperature (30 °C). The data presented are mean ± SEM. * denotes P < 0.05, ** denotes P < 0.01 (n = 6 mice per group).

We next assessed the expression of GATA3, T-bet and Foxp3 in the lung and the spleen. In the lung, the Foxp3 and T-bet mRNA levels from the asthma groups was significantly lower than that in the control groups, which was reversed by treating asthma mice with TT (Fig. 4a). The levels of GATA-3 was significantly different between the asthma group and the control group in both tissues in mice treated witheitherST or TT (Fig. 4a,b). However, the T-bet, Gata-3 and Foxp3 in the spleen showed no difference between the asthmatic groups and the control group (Fig. 4b). The results indicate that the TT may promote the development of Treg cells in asthma mice.

## Discussion

To understand the mechanisms of immunological regulation in asthma is of significancein the clinical diagnosis and treatment. In this study, we identified that the thermoneutral housing temperatures changed the airway physiopathology through activation of systemic immunological function in asthmatic mice. The results demonstrated that TT influenced asthmatic mouse immunity via a shift in the Th1/Th2 cell subsets profile from predominant Th2 towards Th1 pattern in the lung when compared to ST-asthma mice. The asthma mice treated with TT were prevented the development of AHR, the influx of eosinophils and oedema in the lungs and OVA-specific IgE in the serum.

It is observed that asthma may be aggravated in cold environments, in which the mechanism remains unclear. The changes of air temperature may influence the aeroallergens types and levels that may exacerbate allergic respiratory diseases^14^. Previousstudies showed that bronchoconstriction was mostly related to loss of heat and water in the airway following hyperpnoea^5^. When exposure to cold air, the increases in the number of granulocytes and macrophages, and induced mucociliary dysfunction in the lower airways of athletes^2^. The bronchial biopsies of winner athletes showed evidences of airway remodeling characterized by a mixed type of eosinophilic and neutrophilic airway inflammation due to repeatedly and strongly exposed to inhalant irritants and allergens^15^. In the present study, we found that TT could attenuate the peribronchiolar eosinophilia and oedema in asthma mice airway compared with that in the ST-asthma mice.The results suggest that the change of ambient temperature may exacerbate the allergic airway inflammation.

Kokolus et.al reported that the antitumor immunity is significantly increased when the mice were housedat 30 °C compared with 20 °C in murine allograft tumor models^7^. Recent reports indicated that the mutation in Foxn1 of nude mice was associated with the higher energy expenditure. Loss of IL4/IL13 signaling in adipose tissue was a specific defect in adaptive macrophages activation for acclimation to cold^9, 16^. In other words, T cell subsets, especially the CD4^+^ cells in BALB/c mice, can mediate the immune regulation of chronic cold stress. Cell culture temperature can influence T-cell immunity in the peripheral tissues^17^. When cells were cultured at 29 °Cor 41 °C, they would experience the temperature stress^18^. Our results are in line with the reports by showing the change of ambient temperature can alter the imbalance of Th1/Th2 in asthma mice. On the other hand, regulatory T cells (Tregs) play a critical role in suppressing the host immune response, but whether Treg cells are involved in temperature-induced bronchoconstriction is unknown. Here, we found that TT-dependent upregulation of Treg cells in the lung modulated the immune responses in asthmatic mice, and this was correlated with the redressing imbalance of Th1/Th2 cells. Recent researches also highlighted that heat condition could attenuate immune responses through increasing the expression of Foxp3 in the local tissues of tumor microenvironment and autoimmune diseases^9, 19, 20^. Recent reports indicatedthat the impairment of pulmonary CD4^+^CD25^+^ Treg cells might influencethe Th cell balance in asthma^21–25^. These findings suggested that the pulmonary CD4^+^CD25^+^FoxP3^+^Treg cells might contribute to redressing imbalance of Th1/Th2 subsets to the cold stress in asthma.

In conclusion, our study demonstrated that the change of ambient temperature may be an important factor in the pathogenesis of asthma. Our data propose the change of ambient temperature is probably involved in pulmonary Treg cell regulation.

## Methods

### Experiment grouping of mice and ambient temperature conditions

Female BALB/c mice (6–8 week old) were purchased from the Guangdong Medical Laboratory Animal Center (Guangdong, China). The mice were randomly assigned into 4 groups: ST-control, ST-asthma and TT-control, TT-asthma (n = 6). ST-control and ST-asthma groups mice were placed in a climatic box (Huangshi Henfeng Medical Instrument Co. Ltd., Hubei, China) and maintained at the standard temperature (ST) of 20 °C. TT-control, TT-asthma groups mice were placed in another climatic box at the thermoneutral temperature (TT) of 30 °C. The environmental conditions were maintained according to the standard specific pathogen-free animal living conditions of relative humidity (60% ± 10%) and photoperiod (12 h light/dark cycle). Animals were fed on a standard pelleted diet, and sterilized water was provided ad libitum. The study was approved by the Ethics Committee of Guangzhou Medical University, and all procedures were conducted in accordance with the experimental animal guidelines of Guangzhou Medical University.

### Sensitization and airway challengeprocedure

The experimental asthma model was generated as previously described^26^. Figure 1a showed a schematic illustration of the procedures. Ovalbumin (OVA; Sigma Aldrich, St Louis, MO) was mixed with aluminium hydroxide (Sigma Aldrich, St Louis, MO). Mice were injected intraperitoneally (IP) with the mixture containing 20 μg OVA and 4 mg Alum per mouse on day 0 and day 14. On day 21,22 and23, the sensitized mice were challenged with a 1% OVA-in-saline (1 mg/ml) aerosol delivered using an ultrasonic nebulizer (UltraNebs, DeVilbiss, Somerset, PA, USA) for 30 min daily. The mice were sacrificed next day and sampled as described below. The control groups were sensitized and challenged with sterile saline.

### Measurement of airway hyperresponsiveness (AHR)

AHR was measured by recording the responsiveness to methylcholine (Mch) in conscious, unrestrained mice using a whole-body noninvasive plethysmograph (Buxco Electronics, Inc. Wilmington, DE, USA) at the coherent living temperatures in the manual climatic box. This system estimates total pulmonary airflow in mice using a dimensionless parameter known as enhanced pause (Penh) as described previously^27^. Pressure differences were used to calculate Penh values, which are a function of the sum of the airflows in the upper and lower respiratory tracts during the entire respiratory cycle. Penh for baseline and for increasing concentrations of aerosolized Mch were determined by exposing mice to nebulized saline for 2 min and then recorded and the averaged Penh values over 3 min. Mch was aerosolized using an ultrasonic nebulizer, and the aerosol was drawn through the chamber at a constant rate for 2 min, after which Penh values were taken for 3 min and averaged.

### Total and differential inflammatory cell counts in BALF

Upon sacrifice, BALF was collected from each mouse for total and differential cell counting. The total cell number was counted by placing 10 μl BALF in a hemacytometer. The BALF was then centrifuged at 3000 × g at 4°C for 10 min to separate the cells from the fluid. The supernatants were collected for cytokine assay, and cell pellets were resuspended in 100 μl 4% formaldehyde for 1 h prior to hematoxylin & eosin (H & E) staining. At least 200 macrophages, eosinophils, neutrophils and lymphocytes in each sample were counted under a microscope.

### Histopathology examination of the lung

A piece of non-lavaged left lung were excised and fixed in 4% neutral-buffered formalin, then embedded in paraffin and cut into 4 μm sections. The sections were stained with hematoxylin and eosin (H&E) for histological evaluations. Three different fields for each sample were assessed under a light microscope at a magnification of ×200. The lung inflammation was semi-scored by histopathological scoring system as shown by Table 2 ^28^.

**Table 2:** Histopathological scoring system used to assess inflammatory change in lung.

Histopathology grade	Perivascular and peribronchiola eosinophilia	Oedema	Epithelial damage
0	Normal	Normal	Normal
1	Lowgrade cell influx, no tissue pathology	Low grade diffuse oedema	Low grade cell loss
2	Low to moderate cell influx, low grade tissue damage	Moderate alveolar and bronchiolar oedema	Low grade cell loss
3	Moderate cell influx, low grade tissue damage	Regional and focal oedema	Moderate cell loss
4	Moderate to high cell influx, marked tissue damage	Pronounced oedema	Moderate cell loss
5	High cell influx, significant tissue pathology	Pneumonic type oedema	Epithelial metaplasia, mucus cell hyperplasia

### The effects of temperature change on cytokine production of spleen cells

The spleens were collected from each mouse upon sacrifice. Splenocytes were isolated from the spleens as described previously^29^. The spleen cells were cultured in duplicates at a density of 4 × 10^6^ cells/mL in RPMI 1640 medium supplemented with 10% fetal bovine serum (FBS; Life Technologies), 1 mM sodium pyruvate, 2 mM L-glutamine, 0.1 mg/mL streptomycin and 100 U/mL penicillin or with OVA (500 μg/mL). The cells were cultured at 29 °C, or 37 °C, or 41 °C, respectively^17, 18^, The culture supernatants were collected to evaluate the levels of IL-4, IL-13 and IFNγ using ELISA kits (see below)^30^.

### ELISA

Equal portions of parenchymal spleen tissue were cut into small pieces and homogenized with a suitable amount of cold PBS on ice. The resulting suspension was sonicated. Then, the homogenates were centrifuged for 5 min at 5000 × g for 10 min at 4 °C. Protein concentration in the supernatant was determined by the BCA assay method (Thermo Scientific Pierce, Waltham, MA, USA). The levels of IL-4, IL-13 and IFN-γ in spleen homogenate, BALF and the culture supernatants, OVA specific IgE in the serum were assessed using commercial ELISA kits from Cayman Chemical Co. (Ann Arbor, MI, USA) according to the manufacturer’s instructions.

### Flow cytometry

Spleen cells were prepared as described above. The cells were surface-stained with fluoresce in isothiocyan (FITC)-conjugated anti-CD4 (BD Pharmingen, San Diego, CA, USA) and PE-conjugated anti-CD25 (eBioscience) to separate T cells. For analysis of the FoxP3, cells were fixed, permeabilized and stained according to the manufacture instruction for FoxP3 staining (PE-Cy5-conjugated anti-mouse/rat FoxP3 staining kit; eBioscience). To analyze intracelluar cytokine production, the cells were cultured for 48 h in the prescence of 2 mM monensin (BD Pharmingen) with 100 ng/ml phorbolmyristate acetate (PMA) (Alexis, Lausen, Switzerland) and 1 mM ionomycin (Alexis). After washed and blocked with Fc-blockade (CD16/32; BD Pharmingen) for 30 min, cells were directly surface-stained with PE-Cy5-conjugated anti-CD3e (BD Pharmingen) and FITC-conjugated anti-CD4. After fixed and permeabilized using the BD cytofix/cytoperm kit (BD Pharmingen), the cells were stained using PE-conjugated anti-IL-4 (BD Pharmingen) or PE-conjugated anti-IFN-γ (BD Pharmingen). Cells were analyzed (10^4^ gated events were collected) using a FACSCalibur. Background fluorochrome was assessed using appropriate isotype and fluorochrome-conjugated control mAbs. Data collected were analyzed using FlowJo software.

### Total RNA isolation and reverse transcription (RT)-PCR

The total RNA was isolated from tissue using TRIzol according to the manufacturer’s protocol (Invitrogen, Carlsbad, CA, USA) and reverse-transcribed using PrimeScript RT Master Mix (TaKaRa Biotechnology Co., Ltd, Dalian, China). The cDNA was amplified in 96-well reaction plates with a KAPA SYBR FAST Q-PCR kit Master Mix (2×) Universal (KapaBiosystems, Wilmington, DE, USA) in an ABI 7900 real-time PCR thermocycler. PCR cycling conditions were as follows: 95 °C for 3 min and 40 cycles each of 95 °C for 10 s, 56 °C for 20 s, and 72 °C for 10 s. The primer sequences were used as followed: IFN-γ-sense: 5′-AACGCTACACACTGCATCTTGG-3′, IFN-γ-anti sense: 5′-GACTTCAAAGAGTCTGAGG-3′; IL-4-sense: 5′-TCGGCATTTTGAACGAGGTC-3′, IL-4-antisense, 5′-GAAAAGCCCGAAAGAGTCTC-3′; IL-13-sense: 5′-GGAGCTGAGCAACATCACACA-3′, IL-13-antisense: 5′-GGTCCTGTAGATGGT GGC ATT GCA-3′; FOXp3-sense: 5′-TTCATGCATCAGCTCTCCAC-3′, FOXp3-antisense: 5′-CTGGACACCCATTCCAGACT-3′; Gata-3-sense: 5-GGGACATCCTGCG CGAACTG-3, Gata-3-antisense: 5′-CTCCAGCGCGTCATGCACC-3′; Tbet-sense: 5′-GCCAGCCAAACAGAG AAGAC-3′, Tbet-antisense: 5′-ACACACCTCCTACCGACCAG-3′; β-actin-sense: 5′-CCTGTACG CCAACACAGTGC-3′, β-actin-antisense: 5′-ATACTCCTGCTTGCTGATCC-3′. The ΔΔCt- method (change in ΔCt [ = Ct of the target gene minus Ct of the house keeping gene]) was used for relative quantification. Fold changes in expression were calculated according to the transformation: fold increase = 2^(−ΔΔCt)^. PCR efficiency was tested and ensured to be similar for both the target gene and the β-actin control gene.

### Statistical analysis

All analyses were performed with software package SPSS 17.0 (SPSS, Chicago, IL, USA). The data were presented as mean ± SEM. Samples were checked for Gaussian distribution by applying a Kolmogorov-Smirnov normality test. Groups were compared with one-way ANOVA followed by Tukey’s multiple comparison test or two-way ANOVA combined with a Bonferroni post-test. P values < 0.05 were considered statistically significant. All studies were repeated in 3 independent experiments.
